# Predictors of positive esophagogastroduodenoscopy outcomes in children and adolescents: a single center experience

**DOI:** 10.1186/s13104-017-2693-7

**Published:** 2017-07-28

**Authors:** Hernando Lyons, Ying Zhang, Susan Szpunar, Rajmohan Dharmaraj

**Affiliations:** 1 Department of Pediatric Gastroenterology, St. John Hospital and Medical Center, Wayne State University School of Medicine, Detroit, MI 48236 USA; 2 Department of Pediatrics, St. John Hospital and Medical Center, Detroit, MI 48236 USA; 3 Department of Medical Education, St. John Hospital and Medical Center, Detroit, MI 48236 USA; 4 Department of Pediatric Gastroenterology, University of New Mexico, Albuquerque, NM 87106 USA

## Abstract

**Background:**

Esophagogastroduodenoscopy (EGD) has become a key element in the diagnosis and therapy of many gastrointestinal diseases affecting children. The aim of this study was to evaluate predictors of positive outcomes in children undergoing their first diagnostic EGD with biopsies at a single center.

**Results:**

This retrospective study was based on findings from existing EGD and histopathological reports. All procedures were performed between July 2006 and July 2013. Details of each patient’s clinical presentation and EGD were abstracted from medical records to determine the predictors of positive EGD outcomes. A total of 1133 records of patients between the ages of 0 and 18 years old were evaluated. Of these patients, 51.5% (n = 573) were female and 24.5% (n = 278) were younger than 4 years old. The mean age at the time of EGD was 9.6 ± 5.7 years (mean ± standard deviation). The most common indications for the procedure were abdominal pain (54.9%) and emesis (31.9%). The overall prevalence of any endoscopic abnormality was 54.5% and the overall prevalence of any histological abnormality was 59.1%. A multivariate logistic regression found that patients 12 years or older (odds ratio, OR = 1.46; 95% confidence interval, CI 1.31–1.63), African–American race (OR = 2.20; 95% CI 1.45–3.34), dysphagia (OR = 1.96; 95% CI 1.28–3.00) and positive celiac antibodies (OR = 2.25; 95% CI 1.52–3.34) were all significant independent predictors of a positive EGD outcome.

**Conclusions:**

Several clinical variables were found to be independent predictors of positive EGD outcomes in children and adolescents. Prospective studies using standardized definitions of clinical variables and endoscopy outcomes are needed to further understand predictors of positive EGDs.

## Background

Since its inception in the 1960s, the field of pediatric gastroenterology has experienced rapid growth. Pediatric gastroenterology is now an American Board of Pediatrics certified subspecialty that emerged from earlier training of pediatricians in adult gastroenterology units and an increased recognition of gastrointestinal disorders that are unique to children. Over the past 30 years, the number of pediatric gastroenterologists has greatly increased. While there used to be only a few specialists based out of select centers around the world, pediatric gastroenterology is now an ever-growing specialty with approximately one pediatric gastroenterologist per 100,000 children in the United States [1]. With the development of a subspecialty focused on the disorders of the pediatric gastrointestinal tract, new technologies were also developed to aid in diagnoses such as pediatric esophagogastroduodenoscopy (EGD). Pediatric EGD was first introduced in the 1970s [2]. Over the past 30 years, pediatric EGD has evolved from an infrequent procedure performed in the operating room with a monocular viewing of the intestinal lining to a routine outpatient procedure that makes use of intravenous sedation and large viewing screens.

With the increase usage of pediatric EGD procedures the incidence of diseases that require EGD for diagnosis in children has also increased. Franciosi et al. showed that the characteristics of children undergoing EGD, as well as endoscopy practices, changed in the 20-year period from 1985 to 2005 [3]. During this time, there was a 12-fold increase in the number of first-time EGDs performed. This may have also led to an increase in disease incidence rates. An increase of disease rates, however, may instead reflect increasing rates of disease diagnosis rather than a true rise of disease occurrence. The inclusion of children with less severe clinical presentations and the collection of greater numbers of biopsies per procedure might have played a role in increasing rates of disease diagnosis. During this 20-year interval, the number of patients referred for EGD because of gastrointestinal bleeding decreased from 34 to 5%, while the number of patients with abdominal pain increased from 23 to 43%. Additionally, the rate of complete EGD (in which biopsies were taken from the esophagus, stomach, and duodenum) increased from 18% in 1985 to 95% in 2005.

Studies report positive findings in more than 50% of endoscopies performed in children [4–11]. The complication rates associated with these procedures are 1.3% for EGD and less than 1% for colonoscopy [12–18]. These procedures, however, are invasive in that they require intravenous sedation or general anesthesia. Concerns for neurobehavioral disorders and abnormalities in brain function caused by environmental chemical exposure during early brain development have recently been extended to anesthetics and sedatives, which are administered to millions of young children worldwide [19]. They are also associated with significant anxiety for both the patient and the patient’s family [20–23]. Given the invasiveness and anxiety associated with EGD, predictors that could accurately identify children with diseases otherwise only diagnosable by EGD would be useful; however, there is little existing knowledge of such predictors. Thus, the objective of the present study was to identify which predictors could accurately determine the outcomes of diagnostic EGD performed in children and adolescents without known gastrointestinal disease.

## Methods

### Study design

We performed a retrospective chart review based on EGD reports and histopathological findings from the database of the Pediatric Gastroenterology Clinic at St. John Providence Children’s Hospital, a tertiary referral center for the southeast Michigan community. The study was approved by the St. John Hospital and Medical Center Institutional Review Board (IRB) and covered EGDs performed from July 2006 to July 2013. We included patients between the ages of 0 and 18 years old at the time of the procedure. The exclusion criteria for the study were known gastrointestinal disease (foreign body or caustic ingestions, portal hypertension, esophageal stricture, celiac disease, Crohn’s disease, ulcerative colitis or polyp syndromes), a history of bone marrow transplant, cystic fibrosis, Down’s syndrome or previous upper gastrointestinal surgery. Endoscopy examinations performed solely for the placement of feeding gastrostomy catheters were also excluded. To maintain the independence of the endoscopy outcomes, if a child had two or more endoscopies performed during the study period, only the first endoscopy was considered in the analysis. The endoscopy results of children undergoing combined EGD and colonoscopy were excluded. All patients underwent EGD using the pediatric fiber-optic gastroscope at St. John Hospital and Medical Center. All procedures were completed by an experienced team of pediatric gastroenterologists, and all tissue biopsy specimens were reviewed by pediatric pathologists.

Endoscopy findings classified as positive included changes in the mucosa (erythema, friability, edema, nodularity, and atrophy), superficial and deep ulcers, polyps, strictures, varices, and vascular lesions. Positive histology findings included acute or chronic tissue inflammation, presence of infectious agents, polyps, granulomas, lymphangiectasia and metaplasia. Mild inflammation on histology was not considered a positive histology outcome because the clinical significance of isolated mild histology findings is inconclusive [24–26]. For the purpose of our analysis, a positive EGD outcome was defined as any endoscopic or histologic abnormality found (excluding mild inflammation on histology). A single researcher retrieved the following data from each patient’s hospital chart: age, sex, race, presenting symptoms, physical examination findings, laboratory test results, endoscopy abnormalities and histopathology findings. The researcher determined each patient’s top three indications for EGD based on the patient’s record. Categories of indications were abdominal pain, emesis, diarrhea, nausea, regurgitation, choking/gagging, dysphagia, odynophagia, feeding refusal, anorexia, belching, cough, heartburn, bloating/flatulence, throat pain, irritability, weight loss or failure to thrive, positive celiac antibodies of any type and allergies.

### Statistical analysis

Descriptive statistics were generated to characterize the study population. Categorical variables were described as frequencies and continuous variables were described as the mean with standard deviation (SD) or median with interquartile range. The diagnostic yields of the EGD procedures were computed overall and then for each age group. The factors associated with positive EGD findings were assessed using Pearson’s Chi square and Student’s *t* test, as applicable. Any variables found to be significant in the univariate analysis were used as predictors of the probability of abnormal EGD findings in the multivariate logistic regression. All data was analyzed with the statistical package for social science (SPSS) version 23 (SPSS Inc, Armonk, NY, USA) and a p value of 0.05 or less was considered to be statistically significant.

## Results

A total of 1133 endoscopic procedures were performed in a total of 1133 patients during the study period (2006–2013), between the ages of 0 and 18 years old. Of these patients, 51.5% (n = 573) were female and 24.5% (278) patients were younger than 4 years old. The mean age at the time of the procedure was 9.6 ± 5.7 years (mean ± SD). Additional patient characteristics are listed in Table 1. In terms of anesthesia, procedures were performed under oral endotracheal general anesthesia in children younger than 7 years of age, and with a laryngeal mask or natural airway (mask with sevoflurane or nasal cannula with IV propofol) in children aged 7 years and older. There were two major adverse events related to endoscopy itself (1 case of significant bleeding and 1 case of respiratory compromise). The most common primary indications for EGD were abdominal pain (54.9%) and emesis (31.9%). The overall prevalence of any endoscopic abnormality was 54.5%, with findings occurring in the esophagus in 29.2% of patients, stomach 25.2%, and duodenum 13% (patients could have more than one finding). The overall prevalence of any histological abnormality was 59.1%, with pathologic abnormalities occurring in the esophagus in 30.8% of patients, stomach 32.3%, and duodenum 11.6% (patients could have more than one finding). The most common histopathological findings were moderate to severe gastritis (28.2%), moderate to severe esophagitis (21.6%) and mucosal atrophy of the duodenum (8%). The overall prevalence of any histological abnormality was 30.4% in children younger than 4 years of age, 45.3% in children between 4 and 9 years of age, 62.1% in children between 10 and 13 years of age, and 68.6% in children aged 14 years and older. The endoscopic and histological results of the EGDs are summarized in Table 2. EGD revealed a sensitivity of 92%, a specificity of 100%, a positive predictive value of 100%, and a negative predictive value of 89.7%.

**Table 1:** Demographics of study population (n = 1133)

Characteristic	No. (%)
Sex	
Male	540 (48.5)
Female	573 (51.5)
Age (year)	
<4	278 (24.5)
4–9	248 (21.9)
10–13	271 (23.9)
≥14	336 (29.7)
Race	
Caucasian	915 (80.8)
African–American	132 (11.7)
Other	86 (7.5)
Primary indications for EGD^a^	
Abdominal pain	622 (54.9)
Emesis	361 (31.9)
Nausea	258 (22.8)
Failure to thrive/weight loss	177 (15.6)
Allergies	153 (13.5)
Positive celiac antibodies	146 (12.9)
Diarrhea	142 (12.5)
Dysphagia	133 (11.7)
Heartburn	127 (11.2)

*EGD* esophagogastroduodenoscopy

^a^Patients may have had more than one indication for EGD

**Table 2:** EGD abnormalities and histological findings (n = 1133)

Findings^a^	No. (%)
EGD findings	
Esophagus (erythema, erosions, exudates, ulcers)	331 (29.2)
Stomach (erythema, erosions, nodularity, ulcers)	285 (25.2)
Duodenum (erythema, erosions, ulcers)	147 (13)
Histological findings	
Esophagus	
Moderate to severe inflammation	245 (21.6)
Eosinophilic esophagitis	80 (7.1)
Stomach	
Moderate to severe inflammation	319 (28.2)
*Helicobacter pylori* infection	16 (1.4)
Duodenum	
Moderate to severe inflammation	20 (1.8)
Mucosal atrophy	91 (8)

*EGD* esophagogastroduodenoscopy

^a^Patients may have had abnormalities at more than one site

The results of the univariate analysis for predictive factors potentially associated with abnormal EGD are presented in Table 3. The results of the univariate analysis indicated that being age 12 years or older (p < 0.0001), African–American race (p = 0.008), abdominal pain (p = 0.001), choking/gagging (p = 0.001), dysphagia (p = 0.001), feeding refusal (p < 0.0001), coughing (p = 0.004), irritability (p < 0.0001) and positive celiac antibodies (p = 0.001) were all significant predictors of a positive EGD outcome. Multivariate logistic regression found that aged 12 years or older (odds ratio (OR) = 1.46; 95% confidence interval (CI) 1.31–1.63), African–American race (OR = 2.20; 95% CI 1.45–3.34), dysphagia (OR = 1.96; 95% CI 1.28–3.00) and positive celiac antibodies (OR = 2.25; 95% CI 1.52–3.34) were all significant independent predictors of a positive EGD outcome (Table 4).

**Table 3:** Results of univariate analysis for predictive variables potentially associated with a positive EGD outcome compared with a negative EGD outcome (n = 1133)

Predictor variable	Positive EGD outcome: n (%)	Negative EGD outcome: n (%)	p
Age ≥12 year	332 (29.3)	152 (13.4)	*<0.0001*
Female gender	339 (29.9)	245 (21.6)	0.443
African–American race	95 (8.5)	38 (3.4)	*0.008*
Abdominal pain	402 (35.5)	220 (19.4)	*<0.0001*
Vomiting	205 (18.1)	156 (13.8)	0.272
Diarrhea	87 (7.7)	55 (4.9)	0.580
Nausea	165 (14.6)	93 (8.2)	0.073
Regurgitation	59 (5.2)	39 (3.4)	0.822
Choking/gagging	30 (2.6)	43 (3.8)	*0.001*
Dysphagia	97 (8.6)	36 (3.2)	*0.001*
Odynophagia	5 (0.4)	0 (0)	0.062
Feeding refusal	17 (2.5)	33 (7.1)	*<0.0001*
Anorexia	39 (3.4)	30 (2.6)	0.649
Belching	30 (2.6)	29 (2.6)	0.184
Cough	14 (1.2)	24 (2.1)	*0.004*
Heartburn	76 (6.7)	51 (4.5)	0.863
Bloating/flatulence	55 (8.2)	27 (5.8)	0.129
Throat pain	23 (3.4)	16 (3.5)	0.983
Irritability	37 (3.3)	70 (6.2)	*<0.0001*
Weight loss/failure to thrive	101 (8.9)	76 (6.7)	0.541
Positive celiac antibodies	105 (9.3)	41 (3.6)	*0.001*
Allergies	90 (7.9)	63 (5.6)	0.933

Italic values reflect values that are significant

*EGD* esophagogastroduodenoscopy

**Table 4:** Results of multivariate analysis for predictive variables potentially associated with a positive EGD outcome

Predictor variable	p	OR (95% CI)
Age ≥12 year	<0.0001	1.46 (1.31–1.63)
African–American race	<0.0001	2.20 (1.45–3.34)
Dysphagia	0.002	1.96 (1.28–3.00)
Positive celiac antibodies	<0.0001	2.25 (1.52–3.34)

*EGD* esophagogastroduodenoscopy, *OR* odds ratio, *CI* confidence interval

We performed a subgroup analysis based on the different age groups. The patients were categorized by their respective ages at the time of the EGD. The groups were younger than 4, 4–9, 10–13, and 14 years or older (Tables 5, 6). For children younger than 4 years, the results of the univariate analysis revealed that African–American race (p = 0.001) and positive celiac antibodies (p = 0.049) were potential predictors of a positive EGD outcome. Multivariate logistic regression found only African–American race (OR = 3.35; 95% CI 1.67–6.71) to be a significant independent predictor of a positive EGD outcome; for children between 4 and 9 years of age, the results of the univariate analysis revealed that anorexia (p = 0.019), bloating/flatulence (p = 0.028), choking/gagging (p = 0.05) and belching (p = 0.02) were potential predictors of a positive EGD outcome. Multivariate logistic regression found that anorexia (OR = 0.36; 95% CI 0.15–0.84) and belching (OR = 0.27; 95% CI 0.09–0.83) were significant independent predictors of a positive EGD outcome; for children between 10 and 13 years of age, the results of the univariate analysis revealed that the female gender (p = 0.02), diarrhea (p = 0.011) and dysphagia (p = 0.02) were potential predictors of a positive EGD outcome. Multivariate logistic regression found that diarrhea (OR = 13.02; 95% CI 1.54–109.92) and dysphagia (OR = 2.78; 95% CI 1.17–6.60) were significant independent predictors of a positive EGD outcome; for children aged 14 years and older, no predictors were noted in both the univariate and multivariate logistic regression.

**Table 5:** Results of univariate analysis for predictive variables potentially associated with a positive EGD outcome by age

Predictor variable	Age <4 years (n = 278)		Age 4–9 years (n = 248)		Age 10–13 years (n = 271)		Age ≥14 years (n = 336)	
	Positive EGD outcome: n (%)	p	Positive EGD outcome: n (%)	p	Positive EGD outcome: n (%)	p	Positive EGD outcome: n (%)	p
Female gender	41 (14.7)	0.810	81 (32.7)	0.962	75 (27.7)	*0.02*	142 (42.3)	0.076
African–American race	28 (10.1)	*0.001*	20 (8.1)	0.715	14 (5.2)	0.562	33 (10.1)	0.087
Abdominal pain	10 (3.6)	0.305	110 (44.4)	0.352	120 (44.3)	0.550	162 (48.2)	0.255
Vomiting	57 (20.5)	0.477	39 (15.7)	0.968	45 (16.6)	0.925	64 (19)	0.652
Diarrhea	13 (4.7)	0.586	16 (6.5)	0.265	27 (10)	*0.011*	31 (9.2)	0.946
Nausea	1 (0.4)	0.754	36 (14.5)	0.395	50 (18.5)	0.075	78 (23.2)	0.729
Regurgitation	3 (1.1)	0.714	11 (4.4)	0.237	20 (7.4)	0.512	25 (7.4)	0.502
Choking/gagging	20 (7.2)	0.549	1 (0.7)	*0.05*	5 (1.8)	0.746	4 (1.2)	0.576
Dysphagia	3 (1.1)	0.140	17 (6.9)	0.285	32 (11.8)	*0.02*	45 (13.4)	0.100
Odynophagia	0 (0)	–	1 (0.7)	0.430	0 (0)	–	4 (1.2)	0.172
Feeding refusal	13 (4.7)	0.287	2 (0.8)	0.148	2 (0.7)	0.971	0 (0)	–
Anorexia	2 (1.8)	0.559	10 (4)	*0.019*	9 (3.3)	0.782	18 (5.4)	0.474
Belching	2 (0.7)	0.145	5 (2)	*0.02*	8 (3)	0.615	15 (4.5)	0.977
Cough	5 (1.8)	0.783	4 (1.6)	0.077	2 (0.7)	0.091	3 (0.9)	0.141
Heartburn	0 (0)	–	18 (7.3)	0.657	22 (8.1)	0.076	36 (10.7)	0.080
Bloating/flatulence	6 (2.2)	0.774	24 (9.7)	*0.028*	14 (5.2)	0.611	11 (3.3)	0.677
Throat pain	2 (0.7)	0.656	9 (3.6)	0.643	8 (3)	0.941	4 (1.2)	0.515
Irritability	34 (12.2)	0.127	2 (0.8)	0.628	1 (0.4)	0.469	0 (0)	–
Weight loss/failure to thrive	34 (12.2)	0.204	13 (5.2)	0.195	28 (10.3)	0.263	26 (7.7)	0.996
Positive celiac antibodies	11 (4)	*0.049*	35 (14.1)	0.253	36 (13.3)	0.083	23 (6.8)	0.310
Allergies	18 (6.5)	0.155	25 (10.1)	0.751	23 (8.5)	0.808	24 (7.1)	0.323

Italic values reflect values that are significant

*EGD* esophagogastroduodenoscopy

**Table 6:** Results of multivariate analysis for predictive variables potentially associated with a positive EGD outcome by age

Predictor variable	p	OR (95% CI)
Age <4 years		
African–American race	*0.001*	3.35 (1.67–6.71)
Positive celiac antibodies	0.164	0.613 (0.263–1.426)
Age 4–9 years		
Anorexia	*0.018*	0.36 (0.15–0.84)
Belching	*0.022*	0.27 (0.09–0.83)
Choking/gagging	0.098	0.856 (0.714–1.026)
Bloating/flatulence	0.082	1.166 (0.974–1.396)
Age 10–13 years		
Diarrhea	*0.018*	13.02 (1.54–109.92)
Dysphagia	*0.020*	2.78 (1.17–6.60)
Female gender	0.120	0.671 (0.289–1.557)

Italic values reflect values that are significant

*EGD* esophagogastroduodenoscopy, *OR* odds ratio, *CI* confidence interval

## Discussion

The ability to perform diagnostic EGD in adults and children has been one of the defining characteristics of the current era of gastroenterology. It has undoubtedly expanded our understanding of the pathophysiology of common gastrointestinal disorders in children and has been a tremendous tool in the management of patients. As the availability and utility of EGD in the pediatric population has increased over the past three decades, the volume of procedures performed has paralleled that rise. Consequently, decisions regarding the appropriate indications and timing of EGD in children have evolved over time and arguably remain more of an art than a science. More critical review of the use of this tool is needed to maximize efficacy and minimize risk.

This study illustrates the limitations of diagnostic EGD in the workup of many of the most common GI complaints in children. In 55% of cases, there were no EGD abnormalities. The data recorded demonstrated only 30% for histologically confirmed diagnosis in children less than 4 years of age. These findings are similar to those in the few studies reporting EGD outcomes in children and adolescents, and further research is needed to identify patients with the highest risk for treatable etiologies diagnosed by EGD [4–11]. Furthermore, greater discussion is needed to reach a consensus regarding what is an acceptable diagnostic yield for EGD in children and adolescents that takes into account the costs and the risks of the procedure, especially in very young children. Although there will be a wide spectrum of opinions regarding what constitutes an acceptable rate of normal EGD, with greater focus on this issue, quality measures may be established similar to those regarding rates of normal appendectomies in pediatric surgery [27].

In our population of children and adolescents, several clinical characteristics and laboratory variables were found to be independently predictive of positive EGD outcomes, and these predictors differed across the age spectrum. Patients who were older than 12 years of age, African–American race and positive celiac antibodies were significant overall, and these findings are similar to those in the few studies reporting EGD outcomes in children and adolescents; age above 13 years, vomiting, and hypoalbuminemia were significant predictors of positive EGD outcomes [9], and vomiting and duration of symptoms less than 1 year were risk factors for mucosal inflammation in a highly selected group undergoing EGD for symptoms of dyspepsia [28].

Our results are promising and indicate that the clinical utility of predictive factors for positive EGD outcomes merit further exploration. However, we recognize the limitations of a retrospective study. Firstly, the definitions of charted clinical variables and endoscopy findings are not standardized, in that clinician may have a slightly different definition of, for example, diarrhea. This would increase the inconsistency associated with these variables and subsequently increase the sample size required to maintain adequate study power. Secondly, there can be substantial inter-observer variation in identifying mild endoscopic abnormalities such as erythema due to operator experience and the lack of systematic endoscopic assessment. Thirdly, there was no reliable method to control for pre-endoscopy medical treatment, which may have altered the EGD findings, resulting in a discrepancy between a patient’s presenting signs and symptoms and the EGD outcome. Lastly, our database did not identify isolated erythema or other mild abnormalities on EGD. The clinical significance of such findings, similar to isolated mild inflammation on histology, is not clear. Therefore, the inclusion of mild endoscopic abnormalities in the definition of a positive EGD outcome may have resulted in some misclassification of the EGD outcomes.

## Conclusions

In conclusion, in our population of children and adolescents, several predictors were found for positive EGD outcomes. Given the low diagnostic yield of EGD in very young children, we believe that an initial noninvasive workup is reasonable to identify a likely diagnosis prior to any endoscopic evaluation. Prospective studies with standardized definitions of the clinical variables and endoscopy outcomes are expected to improve the clinical significance of EGD prediction.

## Abbreviations

EGD: esophagogastroduodenoscopy
SPSS: statistical package for social science
OR: odds ratio
CI: confidence interval
IRB: Institutional Review Board
